# Activation and Contraction of Human “Vascular” Smooth Muscle Cells Grown From Circulating Blood Progenitors

**DOI:** 10.3389/fcell.2021.681347

**Published:** 2021-08-23

**Authors:** Blerina Ahmetaj-Shala, Isra Marei, Ryota Kawai, Stephen Rothery, Charis Pericleous, Nura A. Mohamed, Hime Gashaw, Kalliopi Bokea, Jake Samuel, Annabelle Vandenheste, Fisnik Shala, Nicholas S. Kirkby, Jane A. Mitchell

**Affiliations:** ^1^National Heart and Lung Institute, Imperial College London, London, United Kingdom; ^2^Department of Pharmacology, Weill Cornell Medicine – Qatar, Doha, Qatar; ^3^Global Project Management Department, Daiichi-Sankyo Co. Ltd., Tokyo, Japan; ^4^Facility for Imaging by Light Microscopy, Faculty of Medicine, Imperial College London, London, United Kingdom; ^5^Department of Biological and Environmental Sciences, Qatar University, Doha, Qatar

**Keywords:** contraction, smooth muscle progenitor cells, calcium, blood vessel, personalized medicine

## Abstract

Blood outgrowth smooth muscle cells (BO-SMCs) offer the means to study vascular cells without the requirement for surgery providing opportunities for drug discovery, tissue engineering, and personalized medicine. However, little is known about these cells which meant that their therapeutic potential remains unexplored. Our objective was to investigate for the first time the ability of BO-SMCs and vessel-derived smooth muscle cells to sense the thromboxane mimetic U46619 by measuring intracellular calcium elevation and contraction. U46619 (10^–6^ M) increased cytosolic calcium in BO-SMCs and vascular smooth muscle cells (VSMCs) but not in fibroblasts. Increased calcium signal peaked between 10 and 20 s after U46619 in both smooth muscle cell types. Importantly, U46619 (10^–9^ to 10^–6^ M) induced concentration-dependent contractions of both BO-SMCs and VSMCs but not in fibroblasts. In summary, we show that functional responses of BO-SMCs are in line with VSMCs providing critical evidence of their application in biomedical research.

## Introduction

Blood vessels consist of smooth muscle lined by endothelial cells. Vascular cells can only be obtained postsurgery or after death. However, both cell types can be grown out and differentiated from human blood (Simper et al., 2002; Sugiyama et al., 2006; Ahmetaj-Shala et al., 2020) in the form of blood outgrowth endothelial cells [BOECs; also known as endothelial colony forming cells (ECFC); Paschalaki and Randi, 2018] and blood outgrowth smooth muscle cells (BO-SMCs; Simper et al., 2002), providing an easily accessible cell source for studying vascular biology in healthy individuals or patients with disease.

The origins of endothelial and smooth muscle progenitor cells that form BOECs and BO-SMCs still remain unclear. For BOECs, initial studies suggested a bone marrow origin but subsequent studies show that they may be derived from tissue vascular niches (Tura et al., 2013; Paschalaki and Randi, 2018). For BO-SMCs, lineage tracing has shown that smooth muscle progenitor cells *in vivo* may form part of the monocyte pool that adhere to activated endothelium and under non-physiological conditions differentiate into smooth muscle cells (SMCs) in the presence of platelet-derived growth factor (Metharom et al., 2008). In line with this, some studies have identified the chemokine CX3CR1 associated with myeloid lineage cells as one of the early markers for smooth muscle progenitor cells (Metharom et al., 2008). It is now increasingly recognized that smooth muscle progenitor cells may also originate from resident cells in the vessel walls (adventitia) (Hu et al., 2004) and/or that endothelial-to-mesenchymal transition may also ultimately lead to SMC phenotypes (Fadini and Tjwa, 2010; Imamura et al., 2010). Currently, BO-SMCs are defined by immunohistochemistry and flow cytometry for typical SMC markers including positive for a-SMA, smooth muscle myosin heavy chain, calponin, and desmin and negative for CD31, CD146, and vascular endothelial cadherin (Simper et al., 2002; Wang et al., 2012; Ahmetaj-Shala et al., 2020). Transcriptomic comparison between BOECs and BO-SMCs show distinct differences in key regulatory pathways including increased expression of platelet derived growth factor receptor pathways, prostaglandin E receptor pathways and interluekin-1 receptor pathways in BO-SMCs (Wang et al., 2012).

In the field of cardiovascular research, BOECs have produced a clear impact and have been used in a number of applications including (i) comparative stem cell phenotyping (Paschalaki and Randi, 2018), (ii) patient phenotyping (George et al., 2014; Paschalaki and Randi, 2018), and (iii) bioassay screens to detect cytokine storm responses (Reed et al., 2015; Ahmetaj-Shala et al., 2020). In contrast, the field of BO-SMCs is in its infancy with, to our knowledge, just 12 primary publications relating to these cells in humans compared with 2,499 identified for BOEC/ECFC (PubMed; July 2021), and it is still unclear if BO-SMCs share functional characteristics with vascular smooth muscle cells (VSMCs). Moreover, BO-SMCs, as with other SMC types, share morphological and cellular similarities with fibroblasts which means that dissociating SMCs from fibroblasts is key but can be challenging. However, while extensive research using these cells in human disease has not yet been conducted, in one study, BO-SMCs were shown to display phenotypic differences in cells from patients with diabetes (van Ark et al., 2012) or Moyamoya disease (Kang et al., 2014) adding evidence to their utility in biomedical research.

A key role of SMCs is to contract and relax in response to physiological stimuli allowing for the homeostatic regulation of blood flow. To date, studies of BO-SMCs have focused on cell biology with just one report demonstrating contraction (Sugiyama et al., 2006). Here, for the first time, we have investigated the ability of BO-SMCs to sense a physiologically relevant stimulus, the thromboxane mimetic U46619, resulting in intracellular calcium elevation and contraction, and (importantly) have directly compared responses to those obtained in vessel-derived SMCs (VSMCs) and fibroblasts. We have selected the ubiquitous contractile agent U46619, which produces a sustained contraction in animal and human blood vessels.

## Methods

### Cell Culture

Human primary pulmonary artery VSMCs and pulmonary fibroblasts were obtained from PromoCell (Germany) and cultured in SMC and fibroblast growth media (5% FBS) respectively, following suppliers’ protocols (PromoCell, Heidelberg, Germany). BO-SMCs were isolated and grown from PBMCs isolated from up to 50 ml whole blood collected in BD Vacutainer Cell Preparation Tubes containing Sodium Heparin/Ficoll (BD Biosciences, Wokingham, United Kingdom). PBMCs and BO-SMCs colonies were maintained in endothelial cell growth media (10% FBS) and emerged as “colonies” which were characterized based on their “hill and valley morphology” as previously described (Ahmetaj-Shala et al., 2020). Since the culture conditions for BO-SMCs and BOECs are identical in this study, it should be noted that, BOECs and/or BOSMCs emerge as colonies which are differentiated from each other by their opposing morphologies. In general terms, one or other of the cell types then emerges as dominant and are passaged/sub-passaged into populations of either BOECs or BOSMCs.

Two separate local ethical approvals were used for blood collection; (i) NRES Committee London—West London and GTAC (15/LO/0223) and (ii) REC Wales approval (17/WA/0161). Male and female (three females; one male) healthy donors were aged between 20 and 45 years. All subjects were healthy volunteers by self-declaration and able to provide written informed consent. Individuals taking any prescription or over-the-counter medicine were excluded from the study. All cells were used between passages 3 and 8. For contraction and calcium studies, cells were plated in glass-bottomed, black-walled 24-well plates (Ibidi, Gräfelfing, Germany) at 1.5 × 10^4^ cells/well and maintained at 37°C in an atmosphere of 95% air/5% CO_2_.

### Calcium Imaging

Cells were incubated in the dark for 40 min with Fluo-4 AM (11 μM)/Powerload^TM^ in extra-cellular solution (ECS; 136 mM NaCl, 5.4 mM KCl, 1 mM MgCl_2_, 0.33 mM NaH_2_PO_4_, 10 mM HEPE, 10 mM D-glucose, and 2.5 mM CaCl_2_). After washing twice with PBS, cells were loaded with ECS and returned to a dark incubator for 30 min to stabilize. U46619 (10^––6^ M) was added to wells and images taken at 0.1- or 0.2-s intervals for 50 s in one field of view. Fluorescence was determined in 10 selected cells using a bespoke ‘‘Intensity’’ ImageJ macro-script^1^ following subtraction of background (*F*_1_–*F*_0_). All images were taken using a Zeiss Axio Observer WF1 or WF3 microscope at ×20 magnification.

### Contraction Studies

Pre-stained cells (Cell Tracker^TM^ Red CMPTX or Green CMFDA dye, Thermo Fisher Scientific, Gloucester, United Kingdom) were maintained in respective media containing low serum (1% FBS, *v*/*v*) for 24 h to facilitate cellular growth arrest. Media was replaced with high glucose DMEM (Sigma-Aldrich, Gillingham, Dorset, United Kingdom) (10% FBS, *v*/*v*) for 1 h prior to imaging. Cells were treated with U46619 given in cumulative concentrations (10^–9^ to 10^–6^ M) at 10-min intervals and images taken every 2 min from two to four selected fields of view per well. Images were inputted into a bespoke ImageJ macro-script to subtract background, enhance contrast, automatically adjust threshold, and track temporal changes in cell surface area (see text footnote 1). Measurements included the initial cell area, length and width. For quantification, all individual cells which were entirely visible (4–10 cells) within the field were chosen from each selected field and the average change in cell size across all cells in each field captured to derive each reported *n*-value.

### Statistics

Data are shown as mean ± SEM and accepted where *p* < 0.05. GraphPad Prism v.9 was used for data analysis. Specific statistical details are shown in the figure legends.

## Results

### BO-SMCs Release Calcium in Response to U46619

**FIGURE 1:**
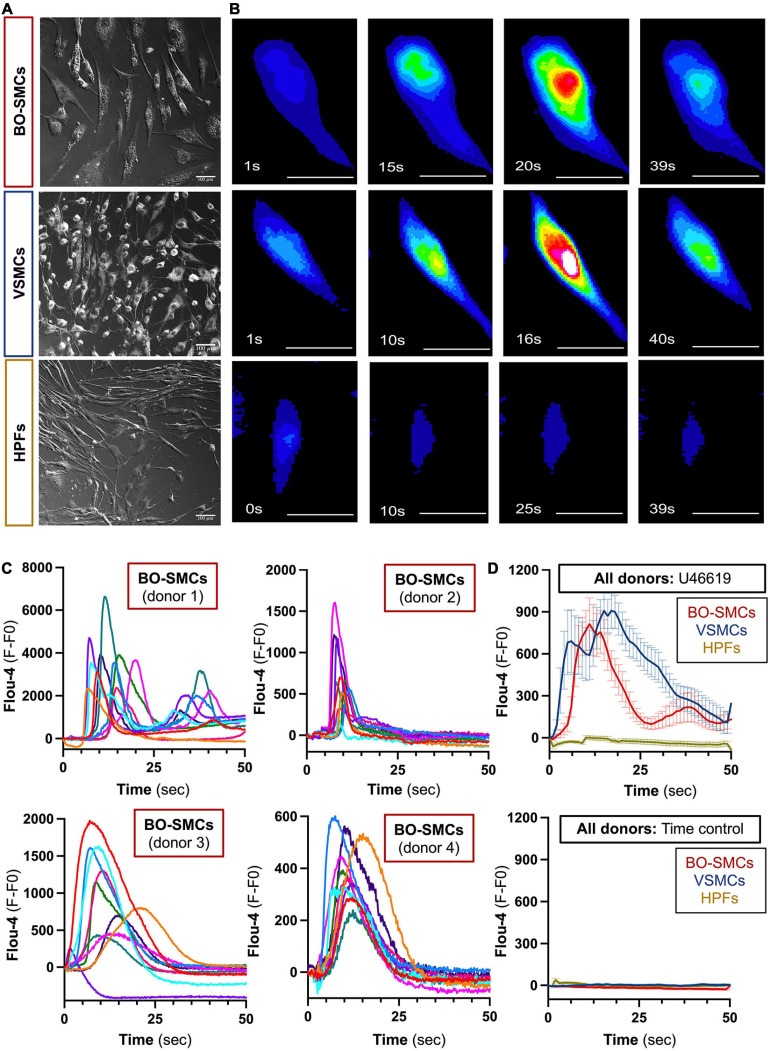
Morphology and comparative calcium responses to U46619 of blood outgrowth smooth muscle cells (BO-SMCs), vascular smooth muscle cells (VSMCs), and fibroblasts (HPFs). Phase contrast images of BO-SMCs, VSMCs, and fibroblasts were obtained using Zeiss Axio Observer WF1 or WF3 microscope at ×20 magnification **(A)**. Represented fluorescence images of cells stained with Flou-4 treated with U46619 (10^–6^ M) **(B)**. Fluorescence intensity (fluorescence minus basal fluorescence taken at *t* = 0; *F*–*F*_0_) tracings from *n* = 10 individual cells per donor imaged at 5–10 frames/s stained with Flou-4 treated with U46619 (10^–6^ M) **(C)**. Pooled data showing mean ± SEM for the average intensity tracings obtained following U46619 (10^–6^ M) treatment (BO-SMCs; *n* = 40 cells comprising 10 individual cells each from four separate donors, VSMCs and HPF; *n* = 30 cells comprising 10 individual cells from three separate donors) or untreated time controls (BO-SMCs; *n* = 30 cells comprising 10 individual cells each from three separate donors, VSMCs, and HPF; *n* = 20 cells comprising 10 individual cells from two separate donors) **(D)**. Scale bars represent 100 μm.

Blood outgrowth smooth muscle cells, VSMCs, and fibroblasts displayed a fusiform morphology (Figure 1A) (length/width ratio, BO-SMCs, 3.32 ± 0.08; VSMCs, 2.66 ± 0.15; fibroblasts, 4.02 ± 0.16; *n* = 307, 154, and 188 cells from 4, 3, and 3 donors, respectively). Under basal conditions, intracellular calcium levels were low and uniform across all cell types (Figure 1 and Supplementary Figure 1). Upon stimulation with U46619, organized changes of cytosolic calcium were observed in BO-SMCs and VSMCs but not fibroblasts. In both BO-SMCs and VSMCs, the initial rise in calcium originated within a defined location consistent with release from the ER from 2 to 10 s after addition of U46619 (10^–6^ M; Figure 1B). Calcium release then spread throughout the cells causing a near global elevation in fluorescence which peaked between 10 and 20 s after U46619 (Figure 1C). Post-peak, the signal declined first in the peripheral areas of the cytoplasm. All VSMC and BO-SMC images showed similar trends in fluorescence after U46619 although there were variations between cells of the same donor and between donors (Figure 1D).

### BO-SMCs Contracted in a U46619 Concentration-Dependent Manner

**FIGURE 2:**
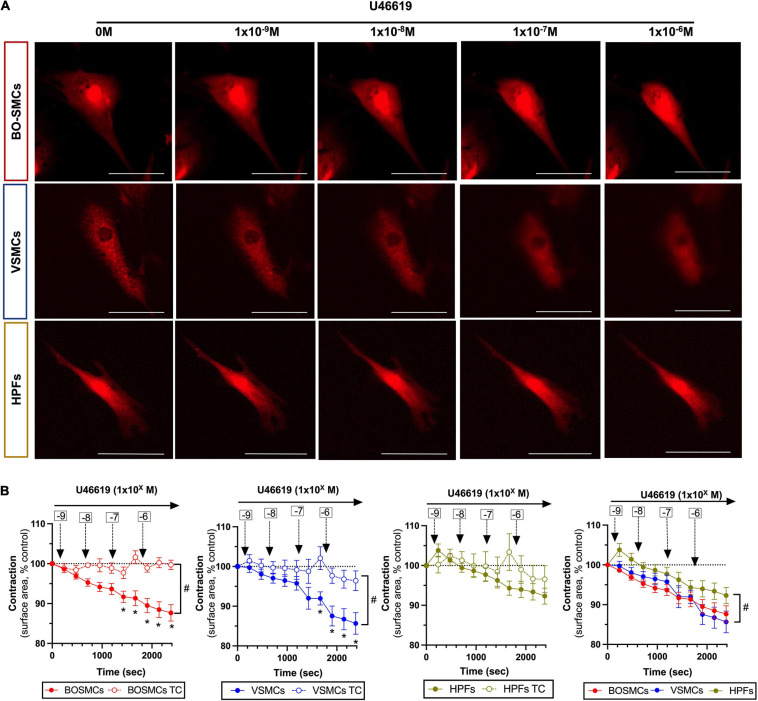
Contraction responses to U46619 of blood outgrowth smooth muscle cells (BO-SMCs), vascular smooth muscle cells (VSMCs), and fibroblasts (HPFs). Representative images of contracting cells captured at 10 min after addition of cumulative concentrations of U46619 (1 × 10^–9^ to 10^–6^ M) **(A)** and pooled data **(B)**. Data in **(B)** are mean ± SEM; for BO-SMCs, *n* = 23 using cells from four separate donors; for VSMCs, *n* = 14 using cells from three separate donors; and for HPFs, *n* = 9 fields using cells from three separate donors. Filled symbols represent treated cells and open symbols represent untreated “time-control” (TC) cells. Statistical differences were tested using two-way ANOVA with a Sidak-multiple comparison *post hoc* test comparing individual U46619 concentration responses with cell-relevant time controls (*p < 0.05) or two-way ANOVA comparing the absence vs. presence of U46619 over time (^#^*p* < 0.05). Data in panel 4 (bottom right hand graph) which include all cell types were tested using a two-way ANOVA (^#^*p* < 0.05) comparing VSMCs or HPFs with BO-SMCs. Scale bars represent 100 μm.

Compared with time control responses, U46619 (10^–9^ to 10^–6^ M) induced significant concentration-dependent contractions of both BO-SMCs and VSMCs, with similar *E*_*max*_ values (87.7 ± 2.1 vs. 85.7 ± 2.7%). By contrast, U46619 had no significant effect on contraction of fibroblasts (Figures 2A,B).

## Discussion

Vascular smooth muscle dysfunction underpins not only cardiovascular disease but also contributes to pathology of a broad range of human diseases including cancer, dementia, and chronic lung diseases. Establishing models to study smooth muscle function using cells from living donors is therefore a critical unmet need. BO-SMCs represent a potential model to address this. However, to date, the field of BO-SMCs has been limited and progress slow due to a lack of function validation.

Fibroblasts can be morphologically similar to SMCs and can be difficult to differentiate from SMCs based on cell markers. Thus, fibroblasts may contaminate cultures of SMCs derived from both vascular and circulating progenitors. With so little known about BO-SMCs, it was important in this study to perform direct functional comparisons with SMCs and fibroblasts. In line with what we know of these cells, fibroblasts and SMCs displayed similar morphologies. However, in our function studies, fibroblasts were relatively unresponsive to activation and the vasoconstrictor actions of U46619. U46619 activates vascular cells *via* cell surface thromboxane, TP receptors and is considered to be a universal contractile agent across a wide range of blood vessels. However, there are other important vasoactive mediators, both contractile (such as endothelin-1, angiotensin, and adrenalin/noradrenalin) and relaxant (nitric oxide and prostacyclin), which underpin vascular homeostasis and as well as disease. The comparative pharmacology of a full range of vasoactive mediators in BO-SMCs is important and will form the basis of future experiments.

## Conclusion

In summary, we present the first proof-of-concept study demonstrating comparative functional contractile and calcium handling in BO-SMCs which confirms their similarity to VSMCs while differentiating them from fibroblasts. This work provides the much-needed corroboration of the usefulness of this novel cell type in vascular biology research which we hope will additionally stimulate the wider vascular community to apply this technology to relevant areas of human cardiovascular disease.

## Data Availability Statement

The raw data supporting the conclusions of this article will be made available by the authors, without undue reservation.

## Author Contributions

BA-S and JAM designed the studies. BA-S, IM, RK, CP, NA, HG, KB, JS, and AV isolated cells and/or collected the data. BA-S, JS, and AV analyzed the data. SR, FS, and NSK provided technical support for data analysis. BA-S and JAM wrote the manuscript. All other authors reviewed and corrected the manuscript where applicable.

## Conflict of Interest

RK was employed by the company Daiichi-Sankyo Co. Ltd. The remaining authors declare that the research was conducted in the absence of any commercial or financial relationships that could be construed as a potential conflict of interest.

## Publisher’s Note

All claims expressed in this article are solely those of the authors and do not necessarily represent those of their affiliated organizations, or those of the publisher, the editors and the reviewers. Any product that may be evaluated in this article, or claim that may be made by its manufacturer, is not guaranteed or endorsed by the publisher.

## Footnotes

1
https://www.imperial.ac.uk/medicine/facility-for-imaging-by-light-microscopy/software/fiji/
