# Supplementary material for: Activation and Contraction of Human “Vascular” Smooth Muscle Cells Grown From Circulating Blood Progenitors
Source: Front Cell Dev Biol. 2021 Aug 23;9:681347. doi: 10.3389/fcell.2021.681347 (PMC8419454; doi:10.3389/fcell.2021.681347)
Supplement: Supplementary file 1 [file Data_Sheet_1.docx]

**Supplementary Figures**

**Supplementary Figure 1:** Calcium response to U46619 of vascular mooth muscle cells (VSMCs) and fibroblasts (HPFs)**.** Fluorescence intensity (fluorescence minus basal fluorescence taken at t=0; F-F_0_) tracings from n=30 cells comprising of 10 individual cells from 3 separate donors ofVSMCs (A) and HPFs (B) imaged at 5-10 frames/sec stained with Flou-4 treated with U46619 (10^-6^M).
